# Supplementary material for: IF1 Promotes Cellular Proliferation and Inhibits Oxidative Phosphorylation in Mouse Embryonic Fibroblasts under Normoxia and Hypoxia
Source: Cells. 2024 Mar 21;13(6):551. doi: 10.3390/cells13060551 (PMC10969582; doi:10.3390/cells13060551)
Supplement: Supplementary file 1 [file cells-13-00551-s001.zip › cells-2812630-supplementary.pdf]

Supplementary Materials:

Western blotting

MEFs were grown in 100 mm dishes and detached via Trypsin/EDTA. Cells were counted, and one million cells were lysed using M-PER Mammalian Protein Extraction Reagent (Thermo Fisher Scientific) as described by the manufacturer. Whole-cell lysates or mitochondria were separated by gradient SDS page (4-15%, Bio-Rad, USA) and transferred onto PVDF membranes (Bio-Rad, USA). Mitochondria were isolated as follow: Cells from one 100mm dish were resuspended in one ml mitochondrial isolation buffer (250 mM sucrose, 0.5 mM Na_2EDTA, 10 mM Tris, all purchased from Sigma-Aldrich, 1% protease, and phosphatase inhibitor Thermo Fisher Scientific, pH 7.4) and transferred into a two ml Dounce homogenizer. Cells were homogenized, and mitochondria were separated by gradient centrifugation. The mitochondrial pellet was washed twice with isolation buffer and resuspended in 50 μL M-PER Mammalian Protein Extraction Reagent.

Protein concentration was determined using a Biotek Synergy HT (Agilent) spectrophotometer as recommended by the manufacturer. Cell lysates were mixed with 4x Laemmli (Bio-Rad, USA) and β-Mercaptoethanol (Sigma-Aldrich) and whole cell lysates were incubated at 95°C for five minutes and mitochondrial samples for 10min on ice before loading. 30 μg of the whole-cell lysates or mitochondria in a separate well containing 10 μL PageRuler™ Prestained Protein Ladder (Thermo Fisher Scientific) were loaded onto a gradient SDS page (4-15%, Bio-Rad, USA) or 15% SDS page gel (for IF1) and separated for 15 min with 50 V and then 100 min at 70 V until the lameli reached the bottom of the gel. The gel was released and then transferred onto PVDF membranes (Bio-Rad, USA) via Trans-Blot Turbo system (Bio-Rad). After the transfer, the following steps were conducted using the Western breeze Chromogenic Immunodetection System (Invitrogen, Thermo Fisher) at room temperature under gentle shaking. Briefly, proteins on the membrane were fixed in 8% Acetic acid in water (Sigma-Aldrich) for five minutes followed by three times washing in water for 5 min. Blocking was conducted for 30min with the provided blocking agent. Membranes were washed twice with water, and primary antibodies were OPA1 (1:1000, Cell signaling, USA), Anti-ATPase Inhibitory Factor 1/IF1 antibody (monoclonal, clone 5E2D7, 1 μg/ml, ab110277, Abcam, UK) and Actin (1:1000, Santa Cruze Biotechnology, USA) incubated for 90min, followed by washing with the antibody washing solution. The secondary antibody solution was incubated for 45 min and washed with antibody washing solution and water. A chromogenic agent (alkaline phosphatase) was added and developed until visible, and the reaction was stopped by washing twice with water. Membranes were air-dried, and images were taken using a ChemiDoc (Bio-Rad).

Quantitative Real-time PCR

MEFs were grown on 100mm dishes, treated with 3 h hypoxia or normoxia as described above, washed, and lysed by adding 1ml Trizol (Sigma-Aldrich). RNA samples were isolated and quantified using the Biotek Synergy HT spectrophotometer. For complementary DNA (cDNA) synthesis, RNA (1μg) was synthesized using a iScript cDNA Synthesis Kit (BioRad) and gradient Mastercycler (Eppendorf, Germany) via the manufacturer's protocol. Quantitative real-time PCR analyses were done using the Step One Real-time PCR system (Applied Biosystems). Expression of IF1 was normalized to β-actin or 36B4, and compared across conditions. Primers for IF1: Fwd: 5′ ATGACAAGATCAAAGATAGCCAAGC 3′; Rev: 5′ GCACAGGAGGAAAGGGAAC 3′. β-actin: Fwd: 5' TTCTGCTTTCCCAGTCATCGTG 3'; Rev: 5′ GAACATCAACCCATTGCTCCC 3′.

Mitochondrial membrane potential

10,000 cells were seeded into black, clear-bottom 96-well plates (Thermo Fisher) and incubated overnight at 37°C and 5% CO2. For assessment, cells were loaded with 50 nM Tetramethylrhodamine methyl ester perchlorate (TMRM) for 30 min at 37°C. The basal membrane potential was read for 5 min followed by an injection of oligomycin (0.5μM final concentration) and was measured for 40 min. In parallel, instead of oligomycin, 25μM FCCP was used to depolarize the mitochondria. For hypoxic conditions, cells were incubated at 2% oxygen as described before for 2.5 hours and then loaded with TMRM as described above followed by the same procedure as with normoxia. TMRM was detected using the Cytation 5 with excitation of 548nm and emission of 574nm. Images were taken every 40 seconds, and they were analyzed using ImageJ. For image data analysis, four fields per well were taken, and fluorescence intensity of at least 20 cells per field were measured.

Supplementary Figures

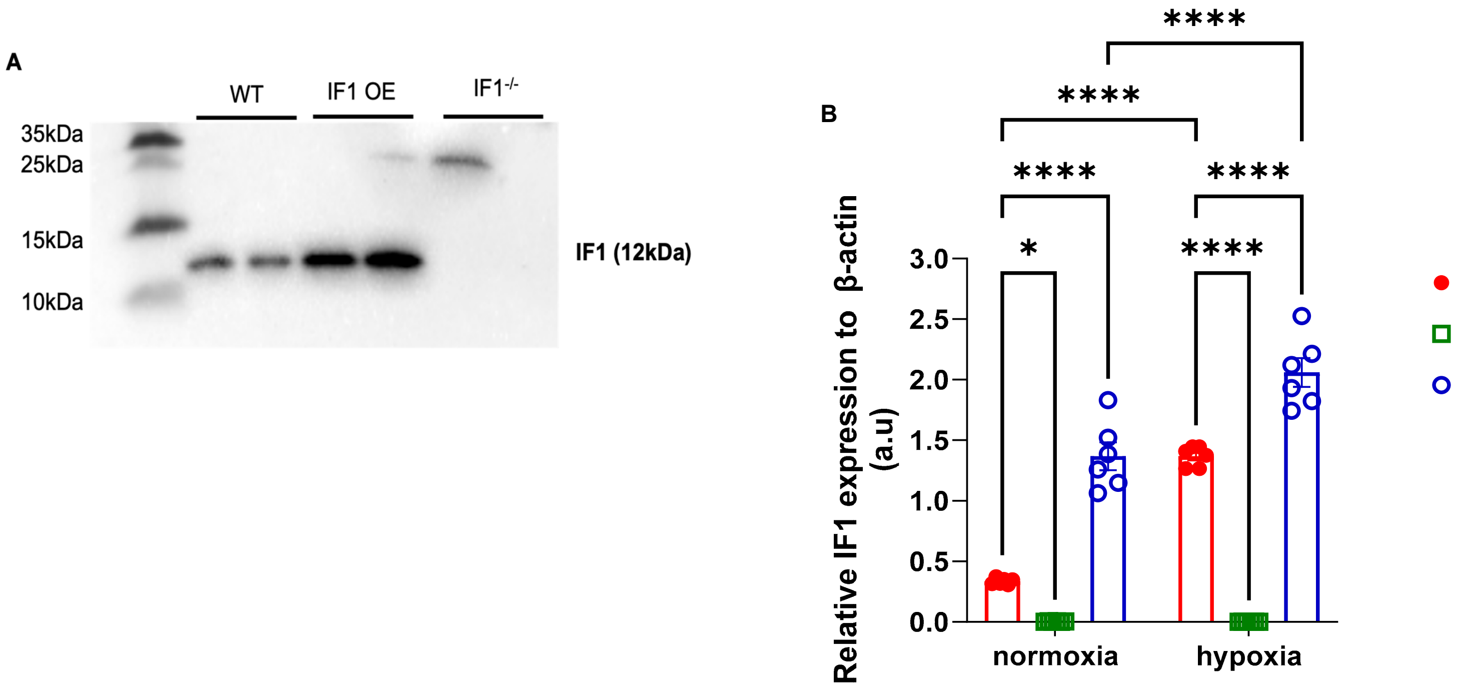

Figure S1: MEFs derived from mice with global IF1 overexpression and knockout. Western blot analyses of IF1 protein expression on total protein extracted from MEFs derived from mice with IF1 overexpression and knockout. MEFs were cultured respectively under normoxia and hypoxia. A) A representative image of western blots. B) IF1 mRNA expression in MEFs with IF1 overexpression and IF1$^{-/-}$ under normoxic and hypoxic conditions. Data are presented as mean ± SEM. *P<0.01; **P<0.001; ***P<0.0001.

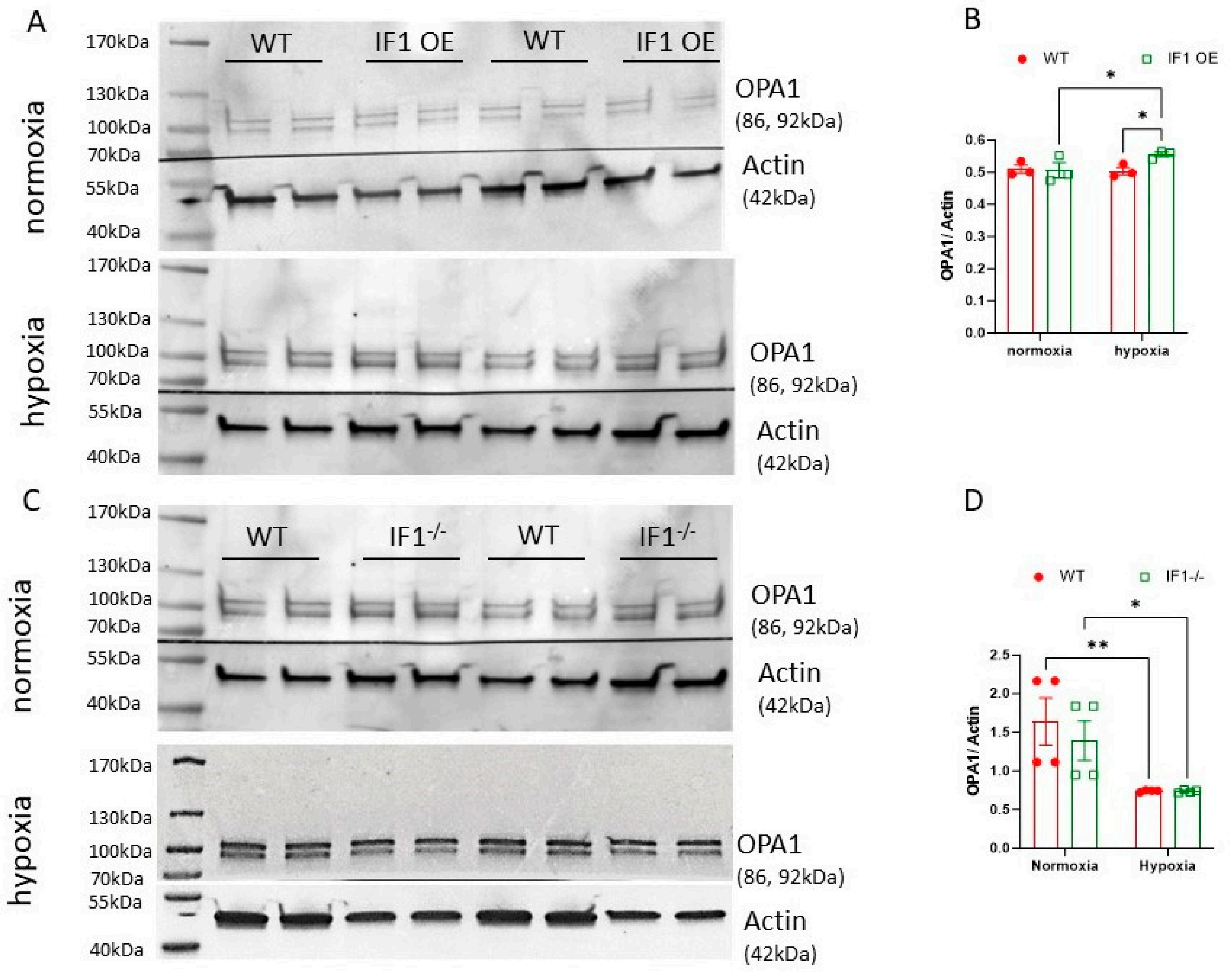

Figure S2: IF1 regulates mitophagy and cristae stabilization under normoxia and hypoxia

A) citrate synthase of mitochondria from wild-type and IF1oe MEF under normoxia and hypoxia. B) mitochondrial copy number from WT and IF1 OE cells during normoxia and hypoxia. C) citrate synthase of mitochondria from control and IF1 KO MEF during normoxia and hypoxia. E) Images of Western blots for OPA1 and B-Actin of WT and IF1oe cells under normoxia and hypoxia. F) analysis of WB. G) Images of Western blots for OPA1 and B-Actin of WT and IF1 KO cells under normoxia and hypoxia. H) analysis of WB. Data are presented as mean ± SEM of three independent experiments. $p < 0.05$ *, $p < 0.01$ **, $p < 0.001$ *** when compared by two-way ANOVA followed by turkey analysis.

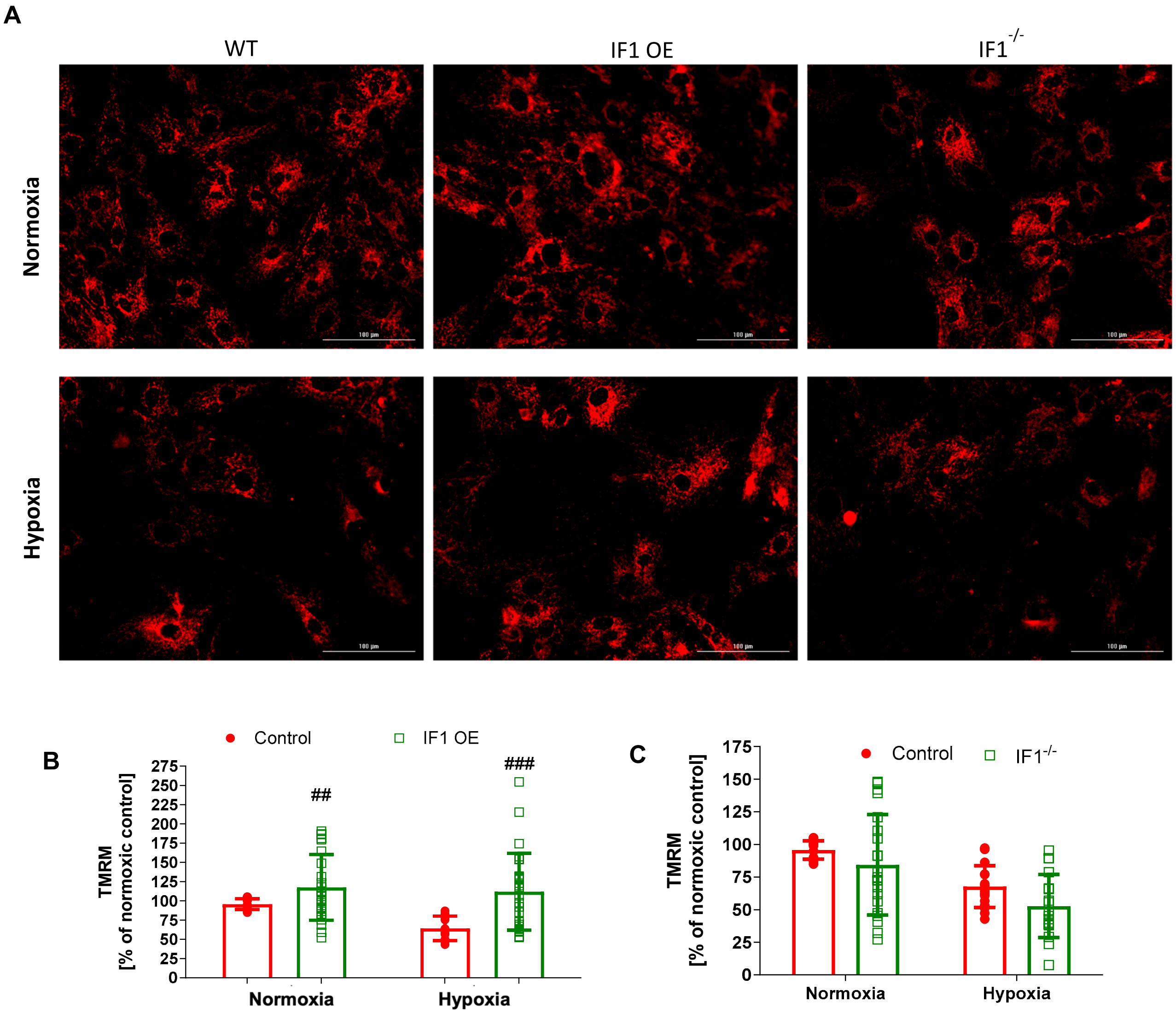

Figure S3: Mitochondrial membrane potential on WT, IF1 OE, and IF1$^{-/-}$ MEFs during normoxia and hypoxia. Mitochondrial membrane potential was measured on TMRM staining MEFs during normoxia and hypoxia. A) Representative images of TMRM-stained MEFs from all experimental groups subjected to normoxia and 3 hours of hypoxia. B) Quantification of TMRM staining intensity in WT and IF1 OE MEFs subjected to normoxia and hypoxia. C) Quantification of TMRM staining intensity in WT and IF1$^{-/-}$ MEFs subjected to normoxia and hypoxia. For image data analysis, four fields per well were taken, and fluorescence intensity of at least 20 cells per field were measured. taken, and fluorescence intensity of at least 20 cells per field were measured. Data is reported as mean ± SEM. Data were analyzed using a two-way ANOVA using Turkey analysis.
